# Carbapenemase Producing *Klebsiella pneumoniae* (KPC): What Is the Best MALDI-TOF MS Detection Method

**DOI:** 10.3390/antibiotics10121549

**Published:** 2021-12-17

**Authors:** Lukáš Hleba, Miroslava Hlebová, Anton Kováčik, Juraj Čuboň, Juraj Medo

**Affiliations:** 1 Institute of Biotechnology, Faculty of Biotechnology and Food Sciences, Slovak University of Agriculture, Trieda Andreja Hlinku 2, 949 76 Nitra, Slovakia; lukas.hleba@uniag.sk; 2 Department of Biology, Faculty of Natural Sciences, University of Ss. Cyril and Methodius in Trnava, Nám. Jána Herdu 577/2, 917 01 Trnava, Slovakia; miroslava.hlebova@ucm.sk; 3 Institute of Applied Biology, Faculty of Biotechnology and Food Sciences, Slovak University of Agriculture, Trieda Andreja Hlinku 2, 949 76 Nitra, Slovakia; anton.kovacik@uniag.sk; 4 Institute of Food Sciences, Faculty of Biotechnology and Food Sciences, Slovak University of Agriculture, Trieda Andreja Hlinku 2, 949 76 Nitra, Slovakia; juraj.cubon@uniag.sk

**Keywords:** *Klebsiella pneumoniae*, KPC, MALDI-TOF MS, detection

## Abstract

*Klebsiella pneumoniae* carbapenemase (KPC)-producing bacteria is a group of highly dangerous antibiotic resistant Gram-negative *Enterobacteriaceae.* They cause infections associated with significant morbidity and mortality. Therefore, the rapid detection of KPC-producing bacteria plays a key role in clinical microbiology. Matrix assisted laser desorption/ionization time-of- flight (MALDI-TOF) is a rapidly evolving technology that finds application in various clinical, scientific, and industrial disciplines. In the present study, we demonstrated three different procedures of carbapenemase-producing *K. pneumoniae* (KPC) detection. The most basic model of MALDI-TOF instrument MS Microflex LT was used, operating in the linear ion-positive mode, commonly used in modern clinical laboratories. The first procedure was based on indirect monitoring of carbapenemase production with direct detection of hydrolyzed carbapenem antibiotic degradation products in the mass spectrum. The second procedure was based on direct detection of *bla*_KPC_ accompanying peak with an 11,109 Da in the mass spectrum of carbapenemase-producing *K. pneumoniae* (KPC), which represents the cleaved protein (pKpQIL_p019) expressed by pKpQIL plasmid. In addition, several unique peaks were detected in the carbapenemase-producing *K. pneumoniae* (KPC) mass spectrum. The third procedure was the identification of carbapenemase-producing *K. pneumoniae* (KPC) based on the protein fingerprint using local database created from the whole mass spectra. By comparing detection procedures, we determined that the third procedure was very fast and relatively easy. However, it requires previous verification of carbapenemase-producing *K. pneumoniae* (KPC) using other methods as genetic *bla*_KPC_ identification, detection of carbapenem degradation products, and accompanying peak with 11,109 Da, which represents cleaved pKpQIL_p019 protein expressed by pKpQIL plasmid. Detection of carbapenemase-producing *K. pneumoniae* using MALDI-TOF provides fast and accurate results that may help to reduce morbidity and mortality in hospital setting when applied in diagnostic situations.

## 1. Introduction

*Klebsiella pneumoniae* is a species of Gram-negative bacteria that belongs to the *Enterobacteriaceae* family [1]. It contaminates different types of food products, including plant raw materials such as sprouts and salads [2], raw vegetables [3], or fresh fruits [4]. It is an important opportunistic pathogen causing frequent nosocomial infections [5], mainly infections of the urinary and respiratory tract [6]. Highly dangerous and rapidly spread strains are represented by carbapenemase-producing *K. pneumoniae* (KPC) often associated with hospital and nosocomial infections, resulting in high morbidity and mortality [7]. Carbapenems are a class of antibiotics that are often used for treatment of Gram-negative bacteria producing extended-spectrum β-lactamases (ESBL) [8]. Carbapenemase-producing *K. pneumoniae* (KPC) and their carbapenemase are classified as class-A, according to Ambler classification of beta-lactamases. Carbapenemase gene is located on plasmids and can hydrolyze all types of penicillins, carbapenems, cephalosporins, and aztreonam [9]. Therefore, the identification of carbapenemase-producing *K. pneumoniae* (KPC) is essential for clinical microbiology mainly. There are several methods for detection of carbapenemase-producing bacteria, such as screening for carbapenemase-production based on disk diffusion methodology using meropenem and ertapenem disks or minimal inhibition concentration [10], combination disk testing [11], biochemical (colorimetric) test [12], carbapenem inactivation method [13], detection of carbapenem hydrolysis with MALDI-TOF MS (matrix-assisted laser desorption/ionization time-of-flight mass spectrometry) [14] or lateral flow assay [15]. These established methods are described in EUCAST guidelines for the detection of resistance mechanisms and specific resistances of clinical and/or epidemiological importance [16]. In addition, newer and faster methods became available in recent years. Today, one of the most common methods used in clinical microbiology is MALDI-TOF MS [17]. The use of MALDI-TOF MS is rapid, robust, and with a low cost per sample. New MALDI-TOF MS approaches for discrimination of bacterial species are emerging recently. They are based on detailed analysis of bacterial proteome by high-resolution mass spectra [18]. Discrimination of microorganisms at the strain level was described in many cases as genetic 16S rRNA sequencing [19], single nucleotide polymorphisms [20], metagenomic sequencing [21], whole-genome sequencing [22], ribosomal multi-locus sequencing [23] or laser induced breakdown spectroscopy and neural networks [24]. Genetic analysis including cell lysis and DNA extraction is time consuming, requires experienced technicians, and is also expensive [22,25,26]. In contrast, MALDI-TOF is easier, faster, cheaper, more accurate, and allows for the identification of closely related microbial strains [27,28,29,30,31]. Moreover, the analysis of unique protein biomarkers in the obtained mass spectrum allows discrimination of bacteria at the strain level [32]. Recently, many applications of MALDI-TOF MS were developed for discrimination of bacteria at the strain level such as discrimination of mycobacteria [33], *E. coli* [32], *Arthrobacter* isolates [34], *Enterococcus faecium* and *Staphylococcus aureus* strains [35], *Bacillus cereus* group [36], highly risk *Escherichia coli* serotypes [37], phylotypes of *Propionibacterium acnes* [38], and many more.

Therefore, the aim of our study was to demonstrate ability of MALDI-TOF MS to distinguish carbapenemase-producing *K. pneumoniae* (KPC) from non-KPC strains. Three identification procedures using MALDI-TOF MS namely (1) hydrolysis of carbapenem antibiotic, (2) specific *K. pneumoniae* (KPC) accompanying peak with 11,109 Da, and (3) building of a local database of spectra for discrimination of both types of *K. pneumoniae* were compared.

Therefore, the aim of our study was to demonstrate ability of MALDI-TOF MS to distinguish carbapenemase-producing *K. pneumoniae* (KPC) from non-KPC strains. Three identification procedures using MALDI-TOF MS namely (1) hydrolysis of carbapenem antibiotic, (2) specific *K. pneumoniae* (KPC) accompanying peak with 11,109 Da, and (3) building of a local database of spectra for discrimination of both types of *K. pneumoniae* were compared.

## 2. Results and Discussion

### 2.1. Verification of Carbapenemase-Producing Klebsiella pneumoniae (KPC)

The disk diffusion methodology showed that all *K. pneumoniae* isolates from small fruits were susceptible to meropenem with a zone diameter higher than 22 mm and 28 mm which represents the screening cut-off value. Only *K. pneumoniae* isolates from patient showed zone diameter smaller than 16 mm. Minimal inhibitory concentration (MIC) was evaluated by MIC Evaluators strips. Klebsiella pneumoniae (KPC) showed resistance against meropenem as MIC values were higher than 8 mg/L and screening cut-off represents 0.125 mg/L. Others *K. pneumoniae* isolates showed susceptibility (≤2 mg/L and smaller than the cut-off value) against meropenem.

Combination disk testing with algorithm for carbapenemase detection was tested against all strains of *K. pneumoniae*. Results confirmed that patient *K. pneumoniae* isolates carried KPC resistance because boronic acid inhibited class A carbapenemase only.

Genetic analysis based on PCR amplification of *bla*_KPC_ genes confirmed presence of the gene solely in the isolates from clinical samples. PCR product with approx. 900 bp was observed on electrophoresis gel. PCR testing showed no amplification for isolates from small berries.

### 2.2. Indirect Detection of Enzymatic Hydrolysis

**Figure 1:**
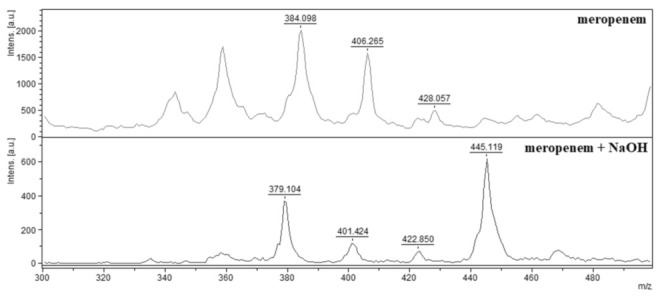
Mass spectra of meropenem and alkaline hydrolyzed meropenem. Meropenem—384.098 *m/z* [M + H]^+^, meropenem sodium salt—406.265 *m/z* [M + Na]^+^, meropenem disodium salt—428.057 *m/z* [M + 2Na]^+^. Alkaline hydrolyzed meropenem: decarboxylated meropenem sodium salt—379.104 *m/z* [M − CO_2_ + H]^+^, meropenem with cleaved amide bond—401.424 *m/z* [M + H]^+^, meropenem sodium salt with cleaved amide bond—422.850 *m/z* [M + Na]^+^ and meropenem disodium salt with cleaved amide bond—445.119 *m/z* [M + 2Na]^+^.

Indirect detection of enzymatic hydrolysis included detection of pure antibiotic (meropenem) as standard, and all degradation products of hydrolyzed meropenem. Meropenem was detected as three peaks complex in the mass spectrum with 384.098 *m/z* (meropenem [M + H]^+^), 406.265 *m/z* (meropenem sodium salt [M + Na]^+^) and 428.057 *m/z* (meropenem disodium salt [M + 2Na]^+^) (Figure 1—meropenem). Similar methodology for carbapenem-resistant *K. pneumoniae* was used by Sakarikou et al. [39] who detected ertapenem by MALDI-TOF MS as three peaks complex: ertapenem with 476.5 *m/z* [M + H]^+^, ertapenem sodium salt with 498.5 *m/z* [M + Na]^+^, and ertapenem disodium salt with 520.5 *m/z* [M + 2Na]^+^. Ertapenem was used for quick *K. pneumoniae* resistance detection also by Yu et al. [40] who observed the same mass spectrum as Sakarikou et al. [39]. Meropenem for indirect detection of enzymatic hydrolysis in *K. pneumoniae* KPC was applied by Wang et al. [41]. The same mass spectrum as in our study was detected. In all these experiments, carbapenem mass spectra were measured in the range from 300 to 500 *m/z*. Most authors used HCCA as matrix because it shows better-quality mass spectra of small molecules than DHB acid. After alkaline hydrolysis, degradation products of meropenem were identified as a mass spectrum with the peaks, as follows: 379.104 *m/z* (decarboxylated meropenem sodium salt), 401.424 *m/z* (meropenem with cleaved amide bond), 422.850 *m/z* (meropenem sodium salt with cleaved amide bond) and 445.119 *m/z* (meropenem disodium salt with cleaved amide bond) (Figure 1—meropenem + NaOH). Alkaline hydrolysis of antibiotics by sodium hydroxide as a comparative model of degradation products was used by all authors in their studies [28,39,40,41,42,43,44].

**Figure 2:**
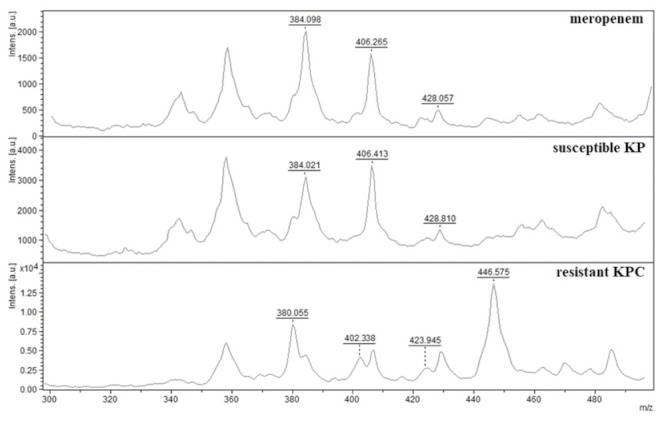
MALDI-TOF mass spectra of meropenem, its sodium salts and degradation products. Meropenem—mass spectrum of meropenem. Susceptible KP—mass spectrum of meropenem in negative control with susceptible *K. pneumoniae*. Resistant KPC—mass spectrum of meropenem degradation products with resistant *K. pneumoniae* KPC. Mass spectra: 384.098 and 384.021 *m/z* (meropenem), 406.264 and 406.413 *m/z* (meropenem sodium salt), 428.057 and 428.810 *m/z* (meropenem disodium salt), 380.055 *m/z* (decarboxylated meropenem sodium salt), 402.338 *m/z* (meropenem with cleaved amide bond), 423.945 *m/z* (meropenem sodium salt with cleaved amide bond) and 446.575 *m/z* (meropenem disodium salt with cleaved amide bond).

Meropenem-resistant and -susceptible isolates of *K. pneumoniae* were incubated in Tris-HCl (pH 7.0) solution supplemented with meropenem. The same solution has been used as buffer by many authors [45,46,47]. Susceptible *K. pneumoniae* did not degrade meropenem and the resulting mass spectrum was identical to that of pure meropenem, described above. In contrast, the mass spectrum obtained from carbapenem-resistant *K. pneumoniae* was the same as the degradation products after alkaline hydrolysis of meropenem. The following peaks were observed: 380.055 *m/z* (decarboxylated meropenem sodium salt), 402.338 *m/z* (meropenem with cleaved amide bond), 423.945 *m/z* (meropenem sodium salt with cleaved amide bond) and 446.575 *m/z* (meropenem disodium slat with cleaved amide bond) (Figure 2). For example, Mirande et al. [44] detected hydrolytic cleavage of ertapenem and faropenem with degradation products, as follows: ertapenem with 450, 472 and 494 *m/z* and faropenem with 304, 326 and 348 *m/z*.

Hrabák et al. [48] hydrolyzed meropenem by carbapenem-resistant bacteria, with Verona imipenemase (VIM) producing *Pseudomonas aeruginosa*, *K. pneumoniae*, *Serratia marcescens,* and *Enterobacter cloacae*, imipenemase (IMP) producing *P. aeruginosa*, KPC *K. pneumoniae*, and New Delhi metallo-β-lactamase (NDM-1) producing *K. pneumoniae*. Subsequently, meropenem trisodium salt with cleaved amide bond (467.839 *m/z*) was detected. In our study, such a degradation product was not observed. Rotova et al. [45] described a method of VIM producing *P. aeruginosa* discrimination based on the detection of hydrolyzed meropenem and meropenem sodium salt [45]. When imipenem is used to detect carbapenemases by MALDI-TOF MS, then it can be also used to detect degradation of carbapenem-resistant species from the OXA-48, KPC, and NDM classes. However, it is not appropriate for the extended-spectrum β-lactamase (ESBL) producers [49]. In our experiment using meropenem, it was possible to identify β-lactamase with an extended spectrum of action (ESBL), which is also confirmed by the results of other authors [48,50]. By detecting degradation products of meropenem, we could confirm the enzymatic hydrolysis of meropenem carbapenem-resistant *K. pneumoniae* KPC. In parallel, a mechanism of resistance commonly called enzymatic destruction of antibiotics has been confirmed. The following table (Table 1) summarizes the relative weights of molecules of native and hydrolyzed or decarboxylated forms of carbapenem antibiotics analyzed by mass spectrometry. For monitoring carbapenem resistance in bacteria it is very important.

### 2.3. Identification of bla_KPC_ Accompanying Peak with 11,109 Da

**Figure 3:**
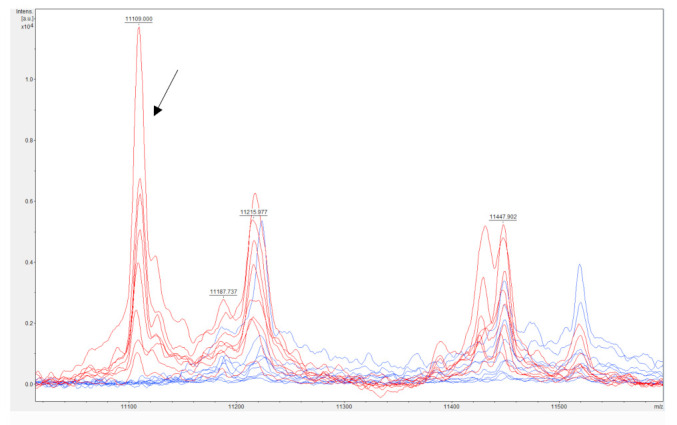
Identification of a peak (indicated by an arrow) associated with *bla*_KPC_ resistance in *Klebsiella pneumoniae* KPC. MALDI-TOF MS spectrum of *bla*_KPC_ positive (red) and negative (blue) *K. pneumoniae*.

The mass spectrum of susceptible and resistant *K. pneumoniae* was obtained using a standard procedure for protein isolation in ethanol-formic acid-acetonitrile extractant. Subsequent crystallization of the sample was performed using HCCA cinnamic acid recommended by Bruker Daltonics and many other researchers [39,52,53,54,55]. The obtained mass spectra were analyzed using flexAnalysis software. In a detailed examination of the mass spectra, the peak in region of 11,109 *m/z* only in carbapenem-resistant *K. pneumoniae* KPCs (marked in red in the Figure 3) was detected. After analysis of *K. pneumoniae* susceptible to carbapenem, the same peak (marked in blue) was not observed. The presence of 11,109 *m/z* peak in spectrum is representing a cleaved protein called pKpQIL_019. Subsequently, the presence of the plasmid pKpQIL, carrying the *bla*_KPC_ gene in carbapenem-resistant *K. pneumoniae* KPC, was detected by PCR. Lau et al. [56] was the first to describe the analysis and discovery of pKpQIL_019 protein by mass spectrometry in relation to carbapenem resistance in *K. pneumoniae* KPC. Presence of plasmid pKpQIL (GenBank Accession No. NC_014016.1), carrying the *bla*_KPC_ gene responsible for carbapenem resistance in *K. pneumoniae* KPC, was confirmed by many researchers [56,57,58,59]. Lau et al. [56] used genetic analysis of *bla*_KPC_ genes, isolation of plasmid pkpQIL, identification of cleavage product of hypothetical protein pKpQIL_019 using Q-TOF LC-MS, subsequent isolation of protein pKpQIL_019, sequencing by N-terminal Edman sequencing, and top-down and bottom-up proteomic analysis that the presence of the pKpQIL_019 hypothetical protein cleavage product in the mass spectrum. Hypothetical pKpQIL_019 protein gene was identified on plasmid pKpQIL next to the Tn4401 transposon between the transposase and resolvase genes [60,61]. The Tn4401 transposon has also been identified in other plasmids [56], specifically in the plasmid IncFIIK [62,63], IncN [64] and ColE [65,66], IncI2 [67,68], and IncX3 [69,70]. A thorough analysis of the pKpQIL sequences in the BLAST databases revealed that the p019 sequence responsible to produce the cleavage product of the hypothetical pKpQIL_019 protein was always identified in the *bla*_KPC_ gene. However, it should be noted that pKpQIL_019 and *bla*_KPC_ are genes located in two different mobile elements. The *bla*_KPC_ gene is located in the Tn4401 transposon and pKpQIL_019 gene between the transposase and resolvase genes. This suggests that it is not entirely possible to associate pKpQIL_019 only with plasmid pKpQIL, but it can be identified as part of the less related plasmids IncFIIK (pKPN-101-IT) [62], IncI2 (pBK15692) [67], or IncX3 (pKPC-NY79) [69].

Cordovana et al. [58] detected the 11,109 *m/z* peak in the mass spectrum of carbapenem-resistant *K. pneumoniae* KPC, which is a cleavage product of the pKpQIL_019 hypothetical protein. They also verified the presence of the *bla*_KPC_ gene and evaluated automated tracking of the 11,109 *m/z* peak in numerous samples. Additionally, they tried to determine stability and repeatability of the 11,109 *m/z* peak detection after culturing *K. pneumoniae* KPC on different nutrient media. Their results showed that the cultivation of *K. pneumoniae* on different nutrient media had no effect on the resulting mass spectrum measured by the MALDI-TOF MS system. The presence of the *bla*_KPC_ gene was confirmed in all cases of the occurrence of a peak with 11,109 *m/z* in the spectrum. Therewith, an automated system for evaluating a large number of samples was successful. As in our experiment, a peak with 11,109 *m/z* in the spectrum was not detected by MALDI-TOF MS in any sample of susceptible *K. pneumoniae*. The 11,109 *m/z* peak was identified solely in isolates with confirmed presence of the *bla*_KPC_ gene. The 11,109 *m/z* carbapenem-resistant *K. pneumoniae* peak can be validated as a suitable marker of KPC producers and considered as appropriate for the detection of carbapenem-resistant *K. pneumoniae* [56,58,59,62,67,69].

**Figure 4:**
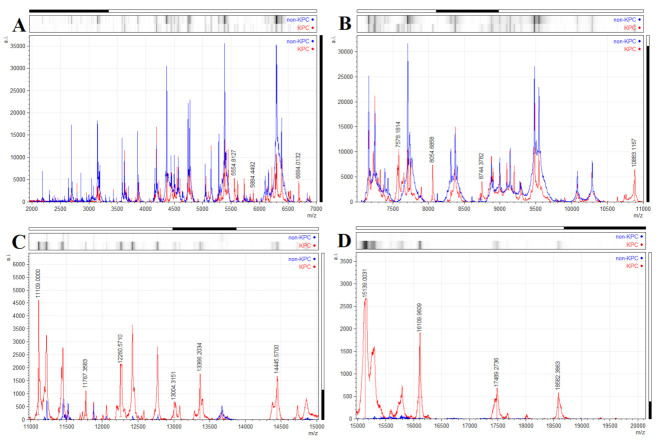
MALDI-TOF MS spectrum of susceptible and resistant *K. pneumoniae* (**A)** from 2000 to 7000 *m/z*, (**B)** from 7000 to 11,000 *m/z*, (**C)** from 11,000 to 15,000 *m/z*, and (**D)** from 15,000 to 20,000 *m/z*. Significantly unique peaks are indicated by an arrow (red—resistant *K. pneumoniae* KPC, blue—sensitive non-KPC *K. pneumoniae*).

In our experiment, a peak with 11,109 *m/z* in the mass spectrum was expected, which is associated with a cleavage product of the protein expressed by the pkpQIL plasmid. The following peaks in the mass spectra of carbapenem-resistant *K. pneumoniae* KPC were detected: 5554, 5884, 6684, 7578, 8054, 8744, 10,883, 11,109, 11,767, 12,260, 13,004, 13,366, 14,445, 15,139, 16,109, 17,489, and 18,582 *m/z*. The peaks mentioned above were absent in the mass spectrum of *K. pneumoniae* susceptible to carbapenem (Figure 4A–D). The exact function of these proteins is not known from the available literature. It is a question for future analyses to document the differences found by mass spectrometry between KPC producers and non-productive strains of *K. pneumoniae* in detail. However, Figueroa-Espinosa et al. [71] observed the predicted enzyme KPC-2 β-lactamase (28,544 *m/z*) in the mass spectrum of KPC-2 production bacterial strains of the family *Enterobacteriaceae*, in particular *K. pneumoniae*, *E. cloacae*, *E. coli*, *Serratia marcescens,* and *Citrobacter braakii*. Additionally, they were able to detect KPC-2 β-lactamase in *P. aeruginosa* KPC-2. However, they found that the presence of the 11,109 *m/z* peak occurs only in KPC-producing strains of *K. pneumoniae* and *E. coli*. Yoon et al. [72] observed significant differences in the range from 28,000 to 29,000 *m/z* after mass spectra analysis of *E. coli* and *K. pneumoniae* strains with KPC-2, KPC-3, and KPC-4 types of β-lactamase resistance. On that basis, they were able to differentiate individual strains. With the help of statistical analyses of the detected mass spectra, they were able to introduce differentiation between KPC production strains by a direct intact method.

### 2.4. Identification Based on the Proteins Fingerprint Database

**Figure 5:**
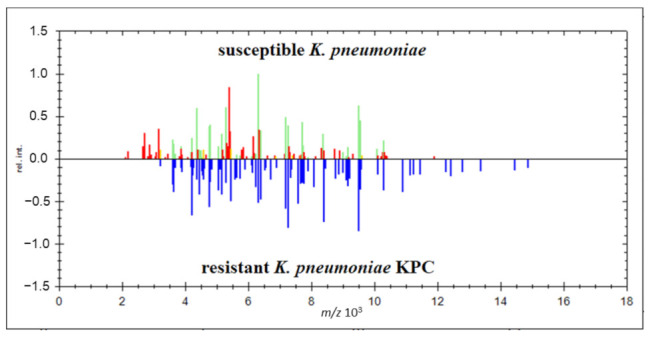
Comparison of mass spectra of susceptible and resistant *K. pneumoniae*.

The most direct and fastest form of carbapenem-resistant *K. pneumoniae* KPC identification may be performed by comparison of the entire mass spectrum. However, it requires the initial creation of a database of mass spectra for resistant and susceptible *K. pneumoniae* types. Standard mass spectrometry procedure was performed using the ethanol-formic acid-acetonitrile extraction method with overlapping and crystallization of the HCCA matrix [39,52,53,54,55]. Spectral maps were created from controlled mass spectra using flexAnalysis software. Using MALDI Biotyper OC software, analyzed spectra of resistant and sensitive *K. pneumoniae* were compared. Significant differences in the mass spectra were found. The identification tool in the MALDI Biotyper software was able to clearly identify resistant and susceptible species of *K. pneumoniae* (Figure 5) with a score higher than 2. Reproducibility reached 100% because all 92 measurements of KPC and non-KPC isolates were correctly identified (Fisher‘s exact test *p* < 0.001). 

**Figure 6:**
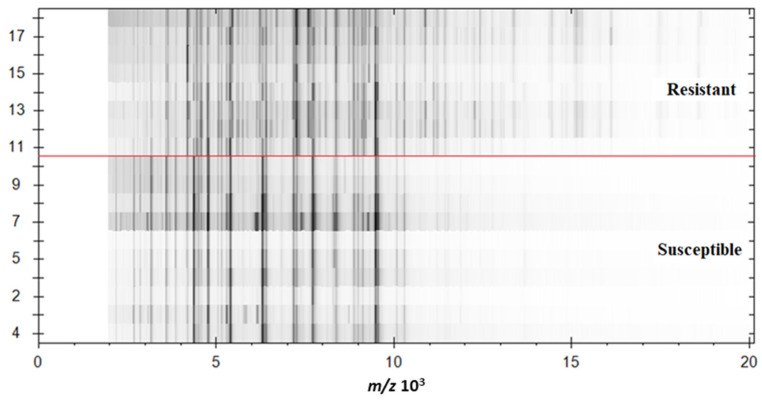
Digital electrophoreogram of resistant and susceptible bacteria *K. pneumoniae* (red line separates resistant from susceptible isolates). Measuring ranges from 2000 to 20,000 *m/z*.

We created a digital electrophoreogram for the discrimination of resistant and sensitive *K. pneumoniae* species stored in a locally created database of mass spectra. MALDI Biotyper subroutine was used to show the protein mass spectrum of resistant and sensitive *K. pneumoniae* isolates. The digital electrophoreogram clearly reads the higher density of detected proteins in the range from 10,000 to 20,000 *m/z* (Figure 6). The results suggested that carbapenem-resistant *K. pneumoniae* showed more peaks representing intact protein in the mass spectrum than susceptible *K. pneumoniae*.

**Figure 7:**
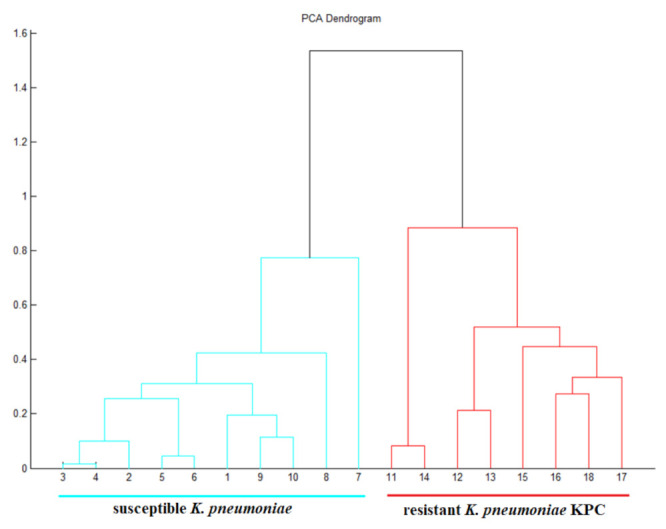
PCA dendrogram showing the relationship of individual samples of susceptible and resistant *K. pneumoniae* isolates based on measured mass spectra.

We clearly defined the boundary between resistant and sensitive species of *K. pneumoniae* by statistical evaluation of mass spectra in the MALDI Biotyper program and subroutine for the creation of PCA dendrograms. The dendrogram showed the relationship between the analyzed *K. pneumoniae* samples based on the obtained mass spectra. The resulting analysis of the PCA dendrogram indicated that the resistant species are classified in the same cluster and the susceptible bacteria *K. pneumoniae* in the other cluster (Figure 7).

Statistical analysis for discrimination of KPC production strains, specifically KPC-2, KPC-3, and KPC-4 was used in work by Yoon et al. [72]. However, they did not analyze the entire mass spectrum obtained in the process of identifying *K. pneumoniae*. They focused only on a specific spectrum ranging from 18,000 to 19,000 *m/z*. The advantage of the technique used in this work is its very simple design. Moreover, it does not require focusing on a specific mass spectrum in KPC. It detects the entire mass spectrum in one step by the automated MALDI-TOF Biotyper system. However, it requires prior preparation of local mass spectra database for KPC resistant and susceptible strains of *K. pneumoniae*. The disadvantage of this technique is the identification of only KPC-producing and non-productive strains of *K. pneumoniae*. We are not able to distinguish individual sub-types of KPC-2, KPC-3 or KPC-4 production strains of *K. pneumoniae* according to the total mass spectrum of *K. pneumoniae*, yet. The question of identifying KPC subtypes of *K. pneumoniae* probably lies on deciphering the unidentified differences in mass spectra, as we described in the chapter above. We were able to detect different mass spectra in KPC production and non-production strains of *K. pneumoniae*. Other more detailed studies need to be performed to confirm or refute our hypotheses.

## 3. Materials and Methods

### 3.1. Bacterial Isolates and Cultivation

Eight clinical isolates of carbapenem-resistant *Klebsiella pneumoniae* (KPC) were obtained from the Slovak Academy of Science, Department of animal physiology, where *bla*_KPC_ gene was detected by PCR method, as described below. Ten Non-KPC isolates were selected from collection of *K. pneumoniae* isolates originated from different types of small fruits as raspberries (*n* = 2), blueberries (*n* = 4) and strawberries (*n* = 4) during years 2019 and 2020. Cultivation of both KPC and non-KPC strains was performed on MacConkey agar (HiMedia, Mumbai, India) for 24 h at 35 ± 2 °C. Pure cultures were obtained from the four-way streak plate method under the same conditions. Pure cultures of KPC and non-KPC strains were stored in sterile glycerol at −80 °C until use.

### 3.2. Identification of Klebsiella pneumoniae

All strains used in this study were identified by MALDI-TOF MS using Microflex LT (Bruker Daltonics, Bremen, Germany) and Biotyper software (Bruker Daltonics, Bremen, Germany). The identification procedure was performed following Hleba et al. [73].

### 3.3. Testing of Klebsiella pneumoniae Carbapenem Resistance

#### 3.3.1. Antimicrobial Susceptibility Testing

Firstly, disk diffusion (Kirby–Bauer) methodology according to EUCAST [74] guideline was performed. Meropenem (MEM) (Oxoid, Hampshire, England) with 10 µg per disk was used in this study. The minimal inhibition concentration (MIC) was tested according to EUCAST [74]. For MIC Evaluator Strips Meropenem (0.002–32 µg/mL) (Oxoid, England) were used. Interpretation of MICs and zone diameter by EUCAST [75] was completed.

#### 3.3.2. Carbapenemase-Production Screening

Screening for carbapenemase production by EUCAST [74] was performed using disk diffusion (Kirby–Bauer) and minimal inhibition concentration methods where meropenem antibiotic was tested as described above [74]. In addition, combination disk testing was used according to EUCAST [16] with the algorithm for carbapenemase detection. Tablets (Rosco, Denmark) containing meropenem with various inhibitors (boronic acid, dipicolinic acid, and ethylenediaminetetraacetic acid) were tested against *K. pneumoniae* isolates. For the elimination of OXA-48 avibactam and AmpC hyperproduction with porin loss, a cloxacillin disk was used. The decision tree method was based on the algorithm for carbapenemase detection established by EUCAST [16]. In addition to the above-mentioned methods, the detection of carbapenem hydrolysis with MALDI-TOF mass spectrometry (Microflex LT, Bruker Daltonics, Bremen, Germany) was used.

#### 3.3.3. KPC Genetic Confirmation

Genomic DNA from each isolate was extracted using EZ-10 Spin Column Bacterial Genomic DNA Miniprep Kit (Biobasic, Markham, ON, Canada). Presence and integrity of DNA was checked on agarose gel and its quantity was estimated using nanophotometer (Implen, Munich, Germany). Concentration of DNA was normalized to 20 ng∙µL^−1^.

The presence or absence of *bla*_KPC_ gene was confirmed by PCR using specific primers [76] which amplify complete sequence of *bla*_KPC_ gene family. Thirty µL of PCR mixture contained Dream Taq Green buffer 1×, 2 mM of DNTP mix, 1 U of DreamTaq polymerase (All chemicals from Thermo, Waltham, MA, USA), 0.5 µM of primer Uni-KPC-F (5′-ATGTCACTGTATCGCCGTCT-3′) 0.5 µM of primer Uni-KPC-F (5′-TTACTGCCCGTTGACGCCC-3′), and 1 µL of extracted DNA.

The amplification was performed using MJ Mini Thermal Cycler (Biorad, Hercules, CA, USA) with the following settings: 95 °C for 3 min for initial denaturation, followed by 35 cycles of 95 °C for 30 s, 55 °C for 30 s and 72 °C for 1 min, and a final extension step at 72 °C for 5 min.

Presence of PCR product was detected using agarose gel electrophoresis (1.5% gel, 1 X TAE buffer, 5 µL DNA loaded). Migration speed was compared to 100 bp plus ladder (Thermo, Waltham, MA, USA) after visualization with ethidium bromide. Isolate was scored as positive for *bla*_KPC_ gene if PCR product of approximately 900 bp was detected.

#### 3.3.4. Indirect Detection of Enzymatic Hydrolysis

**Figure 8:**
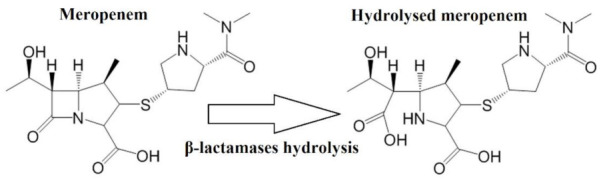
Hydrolysis of meropenem by β-lactamases produced by resistant bacteria.

The main objective of the first procedure was to indirectly detect enzymatic hydrolysis of β-lactams antibiotic meropenem. The principle is based on the hydrolysis of β-lactam antibiotics by β-lactamases produced by resistant carbapenem-producing *K. pneumoniae* (KPC). Subsequently, degradation products of antibiotic were monitored by MALDI-TOF mass spectrometry. Hydrolysis of meropenem using sodium hydroxide was used as a comparative model. Peaks of intact meropenem as well as degradation products were detected by MALDI-TOF and compared to that produced by KPC *K. pneumonia.* Following equation (Figure 8) served as a template of hydrolysis:

#### 3.3.5. MALDI-TOF MS Analysis of Meropenem

In this experiment, commercially available meropenem containing sodium carbonate (meropenem, 500 mg, Hospira UK Limited, Maidenhead, United Kingdom) was used. Different concentrations of meropenem diluted in 20 mM Tris-HCl buffer, pH 7 (Sigma Aldrich, Taufkirchen, Germany) were used for detection by MALDI-TOF MS. Meropenem was diluted in pure MS water 99.9% (Sigma Aldrich, Germany) and pure MS ethanol 99.8% (Sigma Aldrich, Germany) for observation. As a matrix, 2.5 mg α-cyano-4-hydroxycinnamic acid (HCCA) and 2.5 mg dihydroxybenzoic acid (DHB) was used and diluted in 250 mL organic solvent (OR). The organic solvent contained 500 mL of 100% acetonitrile, 475 mL of distilled water, and 25 mL of 100% trifluoroacetic acid (all chemicals were obtained from Sigma Aldrich, Taufkirchen, Germany in MS purity). One microliter of the samples (meropenem in different concentrations diluted in Tris-HCl) was applied on the target plate (Bruker Daltonics, Germany, MSP Target) and allowed to dry at room temperature. Subsequently, the samples were covered with a α-cyano-4-hydroxycinnamic acid (HCCA) matrix. Mass spectra were acquired using Microflex LT mass spectrometer with the FlexControl 3.4 software (Bruker Daltonics, Bremen, Germany) operating in the positive linear ion mode between 300 and 500 *m/z*. Microflex LT using FlexControl software was set in following parameters: ion source 1:20 kV, ion source 2:16.7 kV, lens:7 kV, pulsed ion extraction: 170 ns, detection gain: 3.0×, electronic gain: regular, mode: low range, mass range selection: low range, laser frequency: 60 Hz, digitizer trigger level: 2500 mV, laser attenuator: 24%, laser attenuator: 30%, laser range: 70–90%. Mass spectra by 500 laser shots were measured randomly. The concentration of meropenem was 10 µg/mL. Meropenem was degraded by 100 mM NaOH and diluted in 20 mM Tris-HCl to a final concentration of 10 mM.

#### 3.3.6. Ampicillin and Meropenem Hydrolysis Assay

Purified *K. pneumoniae* bacterial strains were incubated overnight in Mueller-Hinton broth (Biolife, Milano, Italy) to increase the density. One milliliter of high-density inoculum, 4 McF°, was pipetted into the microtube and centrifuged at the maximum speed (13,000 rpm). Pellet was purified using physiological solution that was prepared in 1 mL of 20 mM Tris-HCl buffer (pH 7) with 150 mM NaCl and then centrifuged at the maximum speed for 3 min. The supernatant was discarded. A 50 mL of 50% meropenem diluted in 20 mM Tris-HCl buffer (pH 7) was added to the pellet and mixed by pipette. Afterwards, the resulting mixture was incubated at 35 ± 2 °C for 3 h. After 3 h of incubation, the mixture was centrifuged at the same conditions and 1 mL of supernatant was analyzed by MALDI TOF mass spectrometry.

#### 3.3.7. Analysis of Spectra

For spectrum analysis, flexAnalysis 3.4 software (Bruker Daltonics, Bremen, Germany) was used. Peaks were detected by Centroid detection algorithm with a signal-to-noise threshold of 1, a relative intensity threshold of 0%, a minimum intensity threshold of 0, a peak width of 0.2 *m/z*, a height of 80%, a TopHat baseline subtraction, smoothing with the Savitzky–Golay algorithm, with 0.2 *m/z* width and a one cycle. Theoretical peaks of meropenem, degradation products, and their sodium salts were compared with our detected masses with a ±0.6 *m/z*.

#### 3.3.8. Calibration of MALDI-TOF MS

Calibration of MALDI-TOF MS in this experiment was performed using meropenem, and its degradation products and sodium salts from artificial hydrolysis of meropenem by NaOH.

#### 3.3.9. Identification of *bla*_KPC_ Accompanying Peak with 11,109 Da

The goal was the identification of accompanying peak with 11,109 Da in the mass spectrum of *K. pneumoniae* (KPC), which represents the cleaved protein (pKpQIL_p019) expressed by pKpQIL plasmid. Cleaved protein entitled pKpQIL_p019 is accompanying protein in the expression process of genes of plasmids pKpQIL in carbapenem-resistant *K. pneumoniae* KPC [56].

#### 3.3.10. MALDI-TOF MS Samples Preparing

Intact proteins of *K. pneumoniae* were obtained using standard intact protein extraction procedure established by Bruker Daltonics for identification of microorganisms. Purified overnight cultures were transferred as bacterial colonies to 50 µL solution in microtube containing 25 µL of acetonitrile and 25 µL of formic acid for membrane disruption and obtaining of intact proteins. The resulting suspensions were homogenized by vortexing for cell membrane disintegration. Afterwards, the samples were centrifuged at the maximum speed (13,000 rpm). One µL of supernatant was transferred by pipetting to 96 MALDI-TOF Stainless steel plate (Bruker Daltonics, Bremen, Germany). All samples were overlaid with 1 µL of α-cyano-4-hydroxycinnamic acid (HCCA), which was prepared as previously described. The samples placed on a MALDI-TOF Stainless steel plate were dried at room temperature for crystallization with the matrix.

#### 3.3.11. MALDI-TOF MS Spectrum Obtaining

Mass spectra of *K. pneumoniae* were obtained by MALDI-TOF MS Microflex LT (Bruker Daltonics, Germany) in linear positive mode with a 337 nm pulsed laser. All other settings were performed by Bruker Daltonics standard procedure, which is used for micro-organisms identification in the range from 2 kDa to 20 kDa. Mass spectra were obtained using FlexControl software ver. 3.4 (Bruker Daltonics, Bremen, Germany). 

#### 3.3.12. Mass Spectra Analysis

Mass spectra obtained from all *K. pneumoniae* isolates were analyzed by flexAnalysis software ver. 3.4 (Bruker Daltonics, Bremen, Germany). In this procedure, we focused primarily on the area of the mass spectrum about 11,109 Da, where the accompanying peak should be occurring. In addition, we focused on all differences between resistant and susceptible *K. pneumoniae* along the entire mass spectrum. Determined differences in mass spectra were used for the following differentiation of resistant and susceptible *K. pneumoniae* based on the whole mass spectra saved in our local proteins fingerprint database.

#### 3.3.13. Identification Based on the Proteins Fingerprint Database

The last procedure was to create of proteins fingerprint local database. *Klebsiella pneumoniae* local database was created from the pure, high-quality, and strong intensity (intensity > 1 × 10^3^ arbitrary units—a.u.) mass spectra obtained from a previously described experiment where an accompanying peak with 11,109 Da was acquired. Database included mass spectra of resistant and susceptible *K. pneumoniae* and it was used to compare with the further tested *K. pneumoniae* isolates.

#### 3.3.14. Analysis of Mass Spectra

Mass spectra of all *K. pneumoniae* isolates were analyzed by flexAnalysis version 3.4. We monitored the spectrum from 2 kDa to 20 kDa. Priority was the purity and quality of mass spectra observed by flexAnalysis. We also focused on the intensity of mass spectra, which was at a minimal value of 10^4^. All mass spectra were obtained from minimal 8 replications with 500 laser shots for each sample. The reproducibility of mass spectra was also considered. Obtained mass spectra were transferred to the local database into the MALDI Biotyper OC software version 3.1. Graphical representations of obtained *K. pneumoniae* KPC and non-KPC whole mass spectra were made using MALDI Biotyper OC software ver. 3.1 in the form of an electrophoreogram. mMass software [77] was used for whole mass spectra and differences between KPC and non-KPC spectra of *K. pneumoniae*. The crude spectra were converted from Bruker *.lid files using CompassXport software (Bruker Daltonics, Germany) and forwarded to mMass for processing. All spectra were aligned to the baseline and smoothed by Savitzky–Golay with 0.3 *m/z* and one cycle. The resulting spectra were averaged for better interpretation of the results.

#### 3.3.15. Creation of Local *K. pneumoniae* Database and Verification of Reproducibility

*Klebsiella pneumoniae* local database was created by the same protocol as in our previous study [29]. Reproducibility was verified using a blind test where random isolates of non-KPC and KPC *K. pneumoniae* were chosen and re-analyzed. In four independent time-points, together 28 analyses of KPC isolates and 64 analyses of non-KPC isolates were performed. Analysis was counted as reproducible when MALDI Biotyper OC software correctly identified a sample using our own local database of spectra. Statistical confirmation was obtained by Fisher‘s exact test in Statgraphics XV (Statgraphics Technologies, Inc., The Plains, VA, USA).

## 4. Conclusions

In general conclusion, results confirmed that MALDI-TOF MS is an adequate technique for discrimination of carbapenemase-producing *K. pneumoniae* and non-KPC strain. It should be noted at the outset that all used procedures required purified fresh living bacterial colonies. Comparing of all the procedures used in this experiment led to the following conclusions: The procedure based on observing of hydrolyzed meropenem degradation products required pure bacterial colonies to be cultured overnight, their cultivation with antibiotic, specific set-up of MALDI-TOF for small molecule acquisition ranging from 300 to 500 *m/z*, and the comparison and observation of acquired mass spectrum by post processing mass spectra software. In this case, intact bacterial proteins were not isolated. In addition, the procedure required skilled and experienced technicians. The procedure based on the detection of specific protein biomarker with 11,109 Da, which represent cleaved protein pKpQIL_p019 expressed by pKpQIL plasmid carrying the *bla*_KPC_ gene, required less skilled and experienced technicians, because it is not necessary to set up equipment. It is quite sufficient if the manufacturer’s instructions are followed. Intact proteins can be isolated by standardized procedures and after obtaining the whole mass spectrum, it can be evaluated by post processing mass spectrum software. The operator can easily find the specific peak with 11,109 Da. The last used procedure, based on comparing the whole mass spectrum of *K. pneumoniae* isolates with whole mass spectra saved in created local protein database, is the most rapid and the simplest procedure. This method can also be performed by a less experienced MALDI-TOF MS operator, who is familiar with the basics of identification of microorganisms using mass spectrometry. However, this method required previous confirmation by several methods for detection of carbapenemase-production in *K. pneumoniae* described in this study. The fastest way to identify carbapenem-resistant *K. pneumoniae* (KPC) is a direct identification by the whole mass spectrum with the previous database creation. The limitation lies in the creation of your own database, which is not available yet. Moreover, MALDI-TOF MS requires a large initial investment. However, subsequent analyses are very simple, fast, and inexpensive.

## Figures and Tables

**Table 1:** Native and hydrolyzed forms of carbapenem antibiotics detected by MALDI-TOF mass spectrometry (*m/z*).

C-ATB	Native Forms of ATB (*m/z*)				Hydrolyzed Forms of ATB (*m/z*)			
	[M + H]^+^	[M + Na]^+^	[M + 2Na]^+^	[M + 3Na]^+^	[M + H]^+^	[M + Na]^+^	[M + 2Na]^+^	[M + 3Na]^+^
Meropenem	382 ^1,2,3^	405 ^1,2,3^	427 ^1,2,3^		401 ^2,3^	423 ^2,3^	445 ^2,3^	467 ^2^
Ertapenem	476 ^1,4^	498 ^1,4,5^	520 ^1,4,5^	542 ^1,4^	494 ^1,4^	516 ^1,4^	538 ^1,4^	560 ^4^
Faropenem	286 ^4^	308 ^4^	330 ^4^	352 ^4^	304 ^4^	326 ^4^	348 ^4^	370 ^4^
	Native forms of ATB				Decarboxylated forms of ATB			
	[M + H]^+^	[M + Na]^+^	[M + 2Na]^+^	[M + 3Na]^+^	[M + H_2_O − CO_2_ + H]^+^		[M + H_2_O − CO_2_ + Na]^+^	
Imipenem	300^1^	322^5^			274^5^		296^5^	

C-ATB—carbapenem antibiotics, ATB—antibiotics (^1^ [43]; ^2^ [48]; ^3^ [28]; ^4^ [44]; ^5^ [51]).

## Data Availability

All data are available upon request from the corresponding author.
